# Refractory myasthenia gravis – clinical profile, comorbidities and response to rituximab

**DOI:** 10.3205/000239

**Published:** 2016-10-13

**Authors:** Sreenivasa Rao Sudulagunta, Mona Sepehrar, Mahesh Babu Sodalagunta, Aravinda Settikere Nataraju, Shiva Kumar Bangalore Raja, Deepak Sathyanarayana, Siddharth Gummadi, Hemanth Kumar Burra

**Affiliations:** 1 Columbia Asia Hospital, Kirloskar Business Park, Hebbal, Bangalore, India; 2 Baptist Hospital, Bangalore, India; 3 KS. Hegde Medical College, Mangalore, India; 4 Department of General Medicine, Dr.B.R.Ambedkar Medical College, Bangalore, India

**Keywords:** myasthenia gravis, refractory, rituximab

## Abstract

**Introduction:** Myasthenia gravis (MG) is an antibody mediated autoimmune neuromuscular disorder characterized by fatigable muscle weakness. A proportion of myasthenia gravis patients are classified as refractory due to non responsiveness to conventional treatment. This retrospective study was done to evaluate clinical profile, epidemiological, laboratory, and features of patients with MG and mode of management using rituximab and complications.

**Methods:** Data of myasthenia gravis patients admitted or presented to outpatient department (previous medical records) with MG between January 2008 and January 2016 were included. A total of 512 patients fulfilled the clinical and diagnostic criteria of myasthenia gravis of which 76 patients met the diagnostic certainty for refractory myasthenia gravis and were evaluated.

**Results:** Out of 76 refractory MG patients, 53 (69.73%) patients fulfilled all the three defined criteria. The median age of onset of the refractory MG group was 36 years with a range of 27–53 years. In our study 25 patients (32.89%) belonged to the age group of 21–30 years. Anti-MuSK antibodies were positive in 8 non-refractory MG patients (2.06%) and 36 refractory MG patients (47.36%). Mean HbA_1C_ was found to be 8.6±2.33. The dose of administered prednisone decreased by a mean of 59.7% (*p*=3.3x10^–8^) to 94.6% (*p*=2.2x10^–14^) after the third cycle of rituximab treatment.

**Conclusion:** The refractory MG patients are most commonly female with an early age of onset, anti-MuSK antibodies, and thymomas. Refractory MG patients have higher prevalence and poor control (HbA_1C_ >8%) of diabetes mellitus and dyslipidemia probably due to increased steroid usage. Rituximab is very efficient in treatment of refractory MG with adverse effects being low.

## Zusammenfassung

**Einleitung:** Myasthenia gravis (MG) ist eine neuromuskuläre Autoimmunerkrankung, die durch eine belastungsabhängige Muskelschwäche gekennzeichnet ist. Ein Teil der Myasthenia gravis-Patienten wird als refraktär eingestuft, da sie nicht auf eine herkömmliche Behandlung ansprechen. Die vorliegende retrospektive Studie wurde durchgeführt, um das klinische Profil sowie epidemiologische und Laborwerte von Patienten mit MG zu untersuchen und die Behandlung mit Rituximab und deren Komplikationen zu bewerten.

**Methoden:** Daten von Myasthenia gravis-Patienten, die zwischen Januar 2008 und Januar 2016 aufgenommen wurden oder sich bei ambulanten Behandlungszentren vorstellten (mit vorangegangenen medizinischen Befunden), wurden in die Untersuchung einbezogen. Insgesamt erfüllten 512 Patienten die klinischen und diagnostischen Kriterien von Myasthenia gravis, von denen wiederum bei 76 Patienten sicher refraktäre Myasthenia gravis diagnostiziert werden konnte. Diese Patienten wurden untersucht.

**Ergebnisse:** Von den 76 refraktären MG-Patienten erfüllten 53 (69,73%) alle drei definierten Kriterien. Das mittlere Alter des Auftretens der refraktären MG betrug 36 Jahre mit einem Intervall von 27–53 Jahren. In unserer Studie gehörten 25 Patienten (32,89%) in die Altersgruppe von 21–30 Jahren. MuSK-Antikörper wurden in 8 nicht-refraktären MG-Patienten (2,06%) und 36 refraktären MG-Patienten (47,36%) nachgewiesen. Ein mittlerer HbA_1c_-Wert von 8,6 ± 2,33 wurde festgestellt. Die Dosis des verabreichten Prednison verringerte sich um einem Mittelwert von 59,7% (*p*=3,3×10^–8^) bis 94,6% (*p*=2,2×10^–14^) nach dem dritten Zyklus der Behandlung mit Rituximab.

**Fazit:** Die refraktären MG-Patienten sind am häufigsten Frauen mit einem frühen Krankheitsbeginn, MuSK-Antikörper und Thymomen. Refraktäre MG-Patienten haben eine höhere Prävalenz und eine schlechtere Einstellung (HbA_1c_ >8%) von Diabetes mellitus und Dyslipidämie wahrscheinlich wegen der erhöhten Steroid-Nutzung. Rituximab ist sehr effizient bei der Behandlung von refraktärer MG und weist geringe Nebenwirkungen auf.

## Introduction

Myasthenia gravis (MG) is a relatively rare antibody-mediated neurologic autoimmune disorder and is characterized by fatigable oculobulbar and limb weakness. The word is from Greek µύς “muscle”, αδυναμία “weakness”, and Latin: *gravis* “serious”. It is caused due to formation of antibodies against acetylcholine nicotinic postsynaptic receptors at the neuromuscular junction of skeletal muscles [1], [2]. However, in a rare form, muscle weakness occurs due to a genetic defect in some part of the neuromuscular junction (NMJ) developing through passive transmission from the mother at birth or through autoimmunity later in life that is inherited at birth [3]. The common clinical characteristics of MG are represented in Table 1 (Tab. 1). 

Thomas Willis, Samuel Wilks, Erb, and Goldflam first described myasthenia gravis [4]. In 1895, a German physician named Jolly proposed the term “myasthenia gravis pseudo-paralytica” [4]. Mary Walker treated a patient with MG with physostigmine in 1934 [4]. The autoimmune nature of the condition was described by Simpson and Nastuck [4]. In 1973, Patrick and Lindstrom used rabbits to show that immunization with purified muscle-like acetylcholine receptors caused the development of MG-like symptoms [4].

The estimated annual United States incidence of myasthenia gravis is 2 per 1,000,000. The prevalence of MG in the US ranges from 0.5 to 14.2 cases per 100,000 people. The number of patients increased over the past 2 decades, primarily because of the increased lifespan of patients and also earlier diagnosis [5]. Approximately 15–20% of the patients will experience a myasthenic crisis. Among these patients, around 75% experience their first myasthenic crisis within 2 years of diagnosis [6]. In the United Kingdom, the prevalence of myasthenia gravis is 15 cases per 100,000 population. 

There are no significant studies available in India regarding the prevalence of myasthenia gravis. In Asia, epidemiological studies on Hong Kong Chinese showed a F:M ratio of 2.1:1 [7], [8]. There was a female preponderance. In a hospital-based study from Singapore, myasthenia was noted to be four times more common in Indian males than females although in their ethnic Chinese and Malay patients there was a female preponderance. In Croatia, the prevalence is 10 cases per 100,000 [9]. In Sardinia, Italy, the prevalence increased from 0.75 per 100,000 in 1958 to 4.5 cases per 100,000 in 1986 [10]. 

Myasthenia gravis can occur at any age [11], [12]. Female incidence peaks in the third decade of life, whereas male incidence peaks in the sixth or seventh decade. The mean age of onset is 28 years in females and 42 years in males [11]. Transient neonatal myasthenia gravis occurs in infants of myasthenic mothers who acquire anti-acetylcholine receptor (AChR) antibodies via placental transfer of immunoglobulin G (IgG). Some infants may suffer from transient neonatal myasthenia due to effects of these antibodies [13]. Only 10–20% develop neonatal myasthenic gravis, eventhough most infants born to myasthenic mothers possess anti-AChR antibodies at birth. This may be due to alpha-fetoprotein, which has protective effects inhibiting the binding of anti-AChR antibody to AChR.

High maternal serum levels of AChR antibody increases the risk of neonatal myasthenia gravis. Hence, lowering the maternal serum titer by plasmapheresis during the antenatal period may be helpful. The overall female-to-male ratio has been considered to be 3:2, with a female predominance in younger adults (i.e., patients aged 20–30 years) and a slight male predominance in older adults (i.e., patients older than 50 years) [5], [14]. Studies show that with increased life expectancy, males will be affected at the same rate as females. 

Male preponderance is seen in ocular myasthenia gravis. The male-to-female ratio in children with myasthenic gravis and another autoimmune condition is 1:5. The onset of MG at a young age is slightly common in Asians compared to other races [5]. The clinical classification of the Myasthenia Gravis Foundation of America divides myasthenia gravis into 5 main classes and several subclasses [15], (Table 2 (Tab. 2)). MG is idiopathic in the majority of patients. The end result is the derangement of immune system regulation, eventhough, the cause behind its development is not clearly known. 

In approximately 90% of generalized cases, IgG antibodies to AChR are present [16]. Even in patients not developing clinical myasthenia, anti-AChR antibodies can be demonstrated sometimes. Patients that are found negative for anti-AChR antibodies may be seropositive for antibodies against muscle specific kinase (MuSK). Muscle biopsies in these patients show myopathic signs with mitochondrial abnormalities, as opposed to the neurogenic features and atrophy found frequently in MG patients that are positive for anti-AChR. According to many studies, approximately 85% of patients have antibodies against the nicotinic acetylcholine receptor (nAChR) at the neuromuscular junction [17], [18]. 

The remaining patients have antibodies against other components of the postsynaptic muscle endplate, such as muscle-specific receptor tyrosine kinase (MuSK), or are double seronegative (unidentified or undetected antibody) [18], [19]. The mitochondrial impairment possibly explains the oculobulbar involvement in anti-MuSK-positive MG [20]. Females and patients with certain human leukocyte antigen (HLA) types have a genetic predisposition to autoimmune diseases. The histocompatibility complex profile include HLA-B8, HLA-DRw3, and HLA-DQw2. Systemic lupus erythematosus (SLE) and rheumatoid arthritis (RA) may be associated with MG.

About 10% of patients with generalized MG do not have detectable antibodies to acetylcholine receptor or muscle specific kinase (double seronegative MG). The presence of anti-low density lipoprotein receptor-related protein 4 antibodies (LRP4 abs) has been reported in variable proportion of double seronegative patients. LRP4, a member of the low-density lipoprotein receptor (LDLR) family, contains a large extracellular N-terminal region that possesses multiple epidermal growth factor (EGF) repeats and LDLR repeats, a transmembrane domain, and a short C-terminal region. LRP4 interacts with agrin, and the binding of agrin activates MuSK, leading to the formation of most if not all postsynaptic specializations, including aggregates containing acetylcholine receptors (AChRs) in the junctional plasma membrane. 

Drugs that induce or exacerbate symptoms of myasthenia gravis are represented in Table 3 (Tab. 3). Thymic abnormalities are commonly associated with myasthenia gravis: In MG patients, 75% have thymic disease, 85% have thymic hyperplasia, and 10–15% have thymoma. Extrathymic tumors associated include small cell lung cancer and Hodgkin disease [21], [22]. Hyperthyroidism is present in 3–8% of patients with MG and has a particular association with ocular MG. The term gravis is now a misnomer, as most patients with MG have a near-normal life span due to treatment combining cholinesterase inhibitors, immunosuppressive drugs, plasmapheresis, immunotherapy, and supportive care in an intensive care unit (ICU). 

CD20 is widely expressed on all B cells, i.e., from early pre-B cells to later in differentiation, but is absent on terminally differentiated plasma cells. In humans CD20 is encoded by the *MS4A1* gene. CD20 does not shed, modulate or internalize. Although the function of CD20 is not clearly known, it may play a role in activation of B cells by its function in Ca^2+^ influx across plasma membranes, maintaining intracellular Ca^2+^ concentration. CD20 is the target of the monoclonal antibodies (mAb) rituximab, obinutuzumab, ibritumomab tiuxetan, and tositumomab; all active agents used in the treatment of B cell lymphomas and leukemias. 

The anti-CD20 mAb ofatumumab (Genmab) was approved in October 2009 for chronic lymphocytic leukemia by the FDA. The anti-CD20 mAb obinutuzumab (Gazyva) was approved in November 2013 for chronic lymphocytic leukemia by the FDA. Additional anti-CD20 antibody therapeutics under development include: obinutuzumab for SLE, rituximab for myalgic encephalomyelitis, ocaratuzumab for follicular lymphoma and RA, ocrelizumab for multiple sclerosis (RA discontinued in 2010), TRU-015 (by Trubion) (discontinued in 2010), IMMU-106 (veltuzumab) for non-Hodgkin’s lymphoma or (2015) immune thrombocytopenia. 

Mortality is 3–4% at present, with main risk factors being short history of progressive disease, age older than 40 years, and thymoma; previously, it was as high as 30–40%. A small cohort of patients with generalized MG are refractory to first-line immunotherapies (e.g., azathioprine, cyclosporine, or mycophenolate). Some patients require very high doses of glucocorticoids despite concurrent use of these agents. In these refractory patients, monthly IVIG or the use of rituximab is beneficial. This retrospective study evaluated 76 patients with refractory myasthenia gravis (out of 512 MG patients) to study clinical profile, epidemiological, laboratory data and mode of management, usage of rituximab, complications and prognostic factors. 

## Methods

The retrospective study analyzed data of patients admitted with myasthenia gravis or presented to outpatient department (previous medical records) with MG between January 2008 and January 2016. Data was pooled from 7 hospitals in which 4 are specialized neurological centers. Written and informed consent was obtained from patients to use the data. Consent was obtained from patients during admission or consultation in outpatient basis. Ethical committee approval from the respective hospitals was done. The medical records were analyzed for the demographic data (age, sex), clinical features, co-morbid conditions, investigations, mode and results of the treatment and complications of the procedures. 

A total of 512 patients fulfilled the clinical and diagnostic criteria of myasthenia gravis of which 82 patients met the diagnostic certainty for refractory myasthenia gravis. Six patients were excluded from the study because they did not have all the medical records and consents available. Diabetes mellitus in the patients with refractory MG was diagnosed based on American Diabetes Association (ADA)/WHO criteria (Table 4 (Tab. 4) and Table 5 (Tab. 5)) [23] and previous medical records. Out of 76 patients diagnosed with refractory MG, 22 patients have diabetes mellitus. Out of 76 patients with refractory MG, 42 patients have undergone treatment with rituximab.

All patients studied had a confirmed diagnosis of MG based on the following criteria: 1) Anti-AChR or anti-MuSK antibodies presence in conjunction with either a positive decremental response on repetitive nerve stimulation testing at 3 Hz or a clinical examination consistent with MG or 2) on repetitive nerve stimulation testing at 3 Hz, a positive decremental response in conjunction with a clinical examination consistent with MG and absence of any other disorders that can produce similar clinical features (weakness or fatigue).

Refractory patients were defined as those who could not lower the immunotherapy for MG without clinical relapse, with MG not clinically controlled on their immunotherapy regimen, or who had developed severe adverse effects from immunosuppressive therapy for at least a period of 12 months. There are no clearly defined criteria for refractory MG based on the duration of treatment. We considered a duration of 1 year as relevant. All clinical examinations were supervised by a neurologist. Physical examinations were evaluated before and after rituximab treatment. Rituximab was administered at a standard dose of 375 mg/m^2^. Each cycle is defined as one infusion per week for four consecutive weeks. Interval between cycles was 6 months. 

Conventional immunotherapy used for MG include acetylcholinesterase (AChE) inhibitors (pyridostigmine, neostigmine, and edrophonium), prednisone, plasma exchange (PE), azathioprine, mycophenolate mofetil, cyclosporine and cyclophosphamide; 67 out of 76 patients received prednisolone before taking rituximab; 45 out of 76 patients received PE before taking rituximab. 56 patients were also treated with methotrexate. 

The common indigenous IVIG preparations available in India are Bharat Serum: Ivigama, VHB Pharma: Iviglob, Intas: Globucel, Reliance: Immunorel, Claris: Norglobin, Synergy: MeGlob, and Nirlife Healthcare: IVIG. FDA or EMA preparations of IVIG available in India are Biotest: Intratect, Intraglobin, and Pentaglobin, Bharat Serum/Talecris: Gamunex, Baxter: Kiovig, Gammagard, Novartis: Sandoglobulin, Octapharma: Octagam, and Alpha: Venoglobulin. The average cost per 5 grams of indigenous preparations is around INR 7,500 (US $ 138) while FDA or EMA approved preparations cost around INR 25,000 (US $ 462). The rituximab preparations available in India are Emcure: Ikgdar, Roche: Mabthera, Dr. Reddy’s: Reditux, Roche: Ristova and Mabtas. 

India’s Central Drugs Standard Control Organization (CDSCO) issued a “clarification” to local authorities in 2014 that laboratories intending to engage in bioequivalence and bioavailability (BE/BA) testing must first obtain the approval of the CDSCO before engaging. Subsequent generics of the same drug showing good absorption orally and similar in-vitro release rate need not undergo BA and BE studies, according to the committee. All the drugs administered in the study patients fit in the above guidelines. 

Data analyses were performed using Shapiro-Wilk tests, chi-squared tests, Fischer’s exact tests, and Wilcoxon two-sample tests on SAS and Graph Pad. P-value was considered significant if <0.05. Wilcoxon two-sample test was used to compare the two groups.

## Results

**Figure 1:**
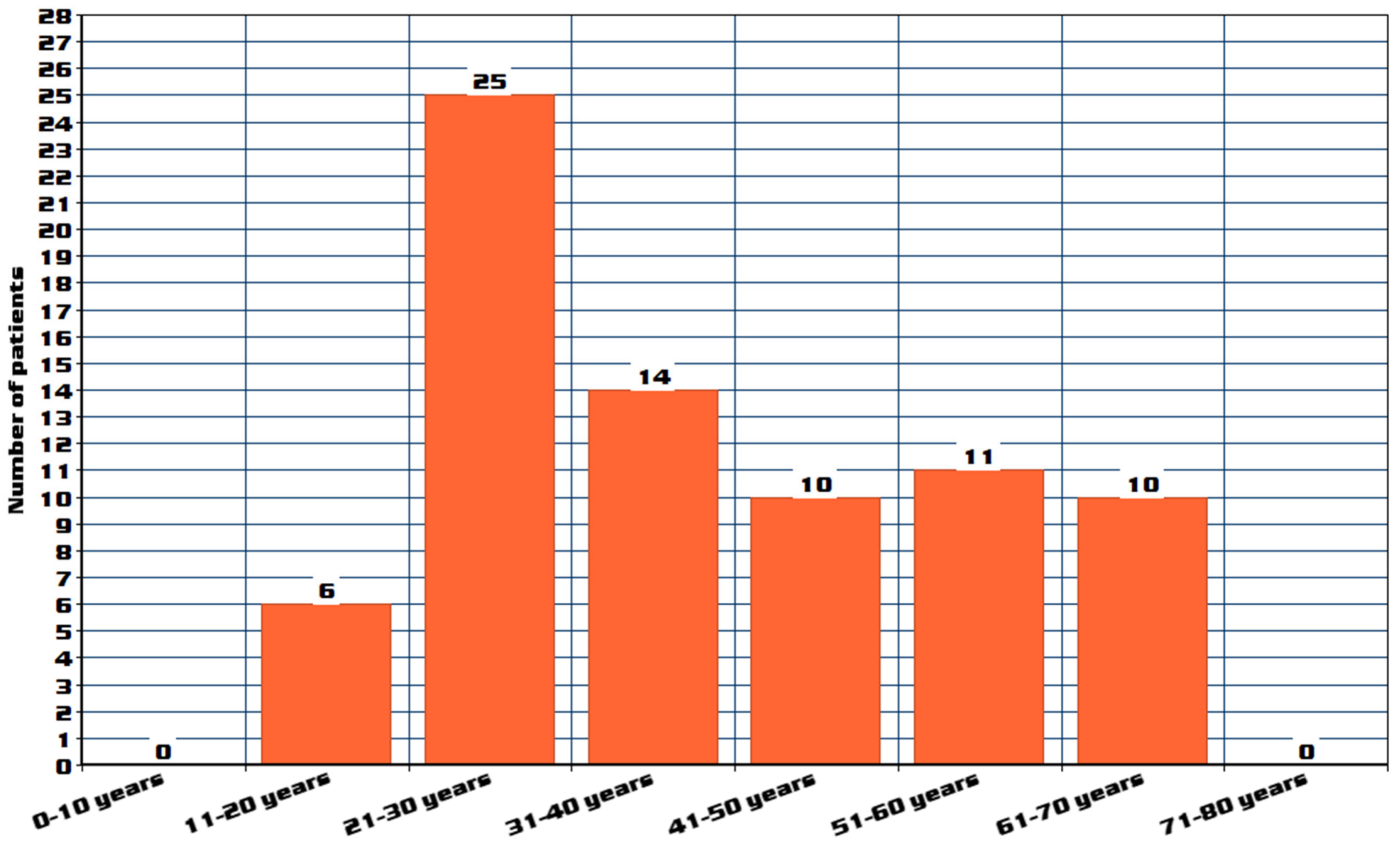
Age distribution of refractory MG patients

A total of 512 patients MG patients were included in the study, out of which 76 patients were classified as refractory MG. Out of 76 refractory MG patients, 53 (69.73%) patients fulfilled all the three defined criteria. Characteristics of refractory MG patients (age of onset, gender, antibody status, previous therapies, and which refractory criteria were met) are represented in Table 6 (Tab. 6). Comparison of refractory MG and non-refractory MG patients is represented in Table 7 (Tab. 7) and Table 8 (Tab. 8). Age distribution is represented in Figure 1 (Fig. 1). The age of onset for MG patients was abnormally distributed according to the Shapiro-Wilk test (p<0.01), with a median age of 56 years (IQR: 38–69) (Table 8 (Tab. 8)). The median age of onset of the refractory MG group was 36 years with a range of 27–53 years. 

The median age of onset of the non-refractory group was 61 years with an IQR of 43–75 years. In our study 25 patients (32.89%) belonged to age group of 21–30 years, followed by 14 patients (18.42%) in 31–40 years group, followed by 11 patients (14.47%) in 51–60 years group, followed by 10 patients (13.15%) in both 41–50 years and 61–70 years groups. A bimodal distribution of age of onset was reported in previous studies, with a peak age below 40 and another peak above 50 years, which correlates with our study [24], [25]. 

The age of onset of MG in the refractory MG group was significantly lower compared to the non-refractory MG group (p<0.001). Out of our total MG patients, 50.97% were female patients. A higher proportion of refractory MG patients were female in comparison with non-refractory MG patients. Fifty-six patients (73.68%) out of 76 refractory MG patients and 205 patients (47.01%) out of 436 non-refractory MG patients were female which is statistically significant (p=0.029).

Regarding anti-AChR and anti-MuSK antibodies, antibody status was found for all 512 patients. But, due to the investigations done outside the hospital and laboratories not accredited by the National Accreditation Board for Testing and Calibration Laboratories (NABL) and the National Accreditation Board for Hospitals & Healthcare Providers (NABH), 48 patients were excluded. Out of 464 patients (90.62%), 330 patients (71.12%) were found positive for anti-AChR antibodies, 44 patients (9.48%) were positive for anti-MuSK antibodies and 90 patients (19.39%) were double seronegative. Among refractory MG and non-refractory MG patients, anti-AChR antibodies were positive in 290 non-refractory MG patients (74.74%) and 40 refractory MG patients (52.63%). 

Anti-MuSK antibodies were positive in 8 non-refractory MG patients (2.06%) and 36 refractory MG patients (47.36%) which is statistically significant (p<0.001). No seronegative patients were found in refractory MG group, while 90 non refractory MG patients (23.19%) were double seronegative(p=0.018). A total of 122 MG patients (23.82%) have undergone thymectomy of which non-refractory MG group consists of 80 patients (18.34%) and refractory MG group consisted of 42 patients (55.26%), which is statistically significant (p<0.001). Thymectomy approaches commonly used are trans-sternal and video-assisted thoracoscopic thymectomy. 

Thymoma status was available for 312 (60.9%) patients of which, 56 (17.94%) patients were found to be thymomatous and 256 (82.05%) patients were non-thymomatous. Among thymomatous, 20 (41.66%) patients of refractory MG was found, while 36 (13.63%) patients of non-refractory MG was found, which is statistically significant (p=0.02). Among refractory MG patients, 22 patients (28.94%) were found to have diabetes mellitus. Among these diabetic patients, 21 (95.45%) patients have been found to have dyslipidemia. All these 22 (100%) patients received prednisolone. 

**Figure 2:**
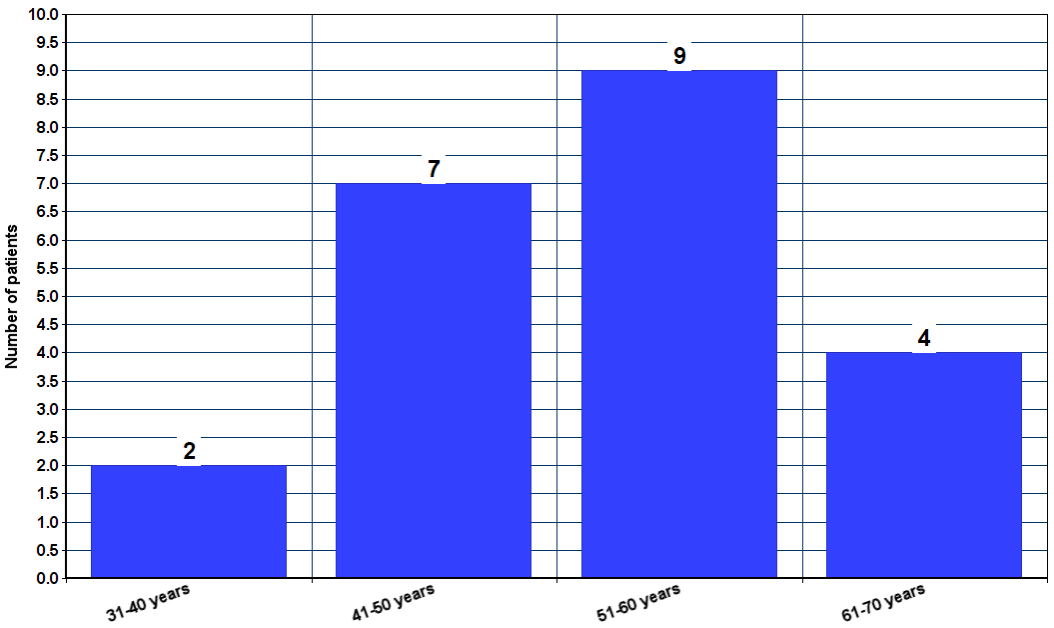
Age distribution of refractory MG patients with diabetes mellitus

Characteristics of these refractory MG patients with relative risk and statistical significance were represented in Table 7 (Tab. 7) and Table 8 (Tab. 8) and characteristics of refractory MG patients with diabetes mellitus (DM) were analyzed in Table 9 (Tab. 9). Age distribution of patients with DM along with refractory MG are represented in Figure 2 (Fig. 2). Females constituted 72.72%, while males constituted 27.27% of refractory MG patients with DM. Mean HbA_1C_ was found to be 8.6±2.33. HbA_1C_ was ≥8% for 68.18% of patients. Only 9.09% of patients have HbA_1C_ below 7%. Hypertriglyceridemia was found in 21 out of 22 DM patients (95.45%). Anti-GAD antibodies and C-peptide levels were in the normal range for all patients. There was no significant difference observed among hypertension, ischemic heart disease and thyroid disorders between refractory MG and non-refractory MG groups. 

Among refractory MG patients, 42 patients received rituximab as treatment. Rituximab was given at a standard dose of 375 mg/m^2^, with each cycle defined as 1 infusion per week for 4 consecutive weeks. Interval between cycles was 6 months. Among the patients that received rituximab, 39 patients received prednisolone, 36 patients received plasma exchange, and 3 patients received mycophenolate mofetil. Regarding rituximab infusions, 18 patients received 2 infusions, while 24 patients received three or more than three infusions of rituximab. 

**Figure 3:**
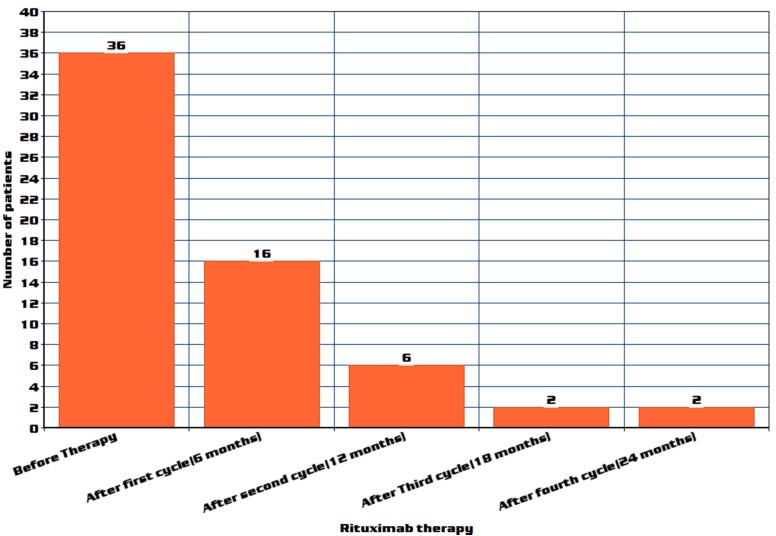
Plasma exchange reduction in number of patients after rituximab therapy

All 39 patients on prednisone treatment showed a dose reduction, after cycle 3, three patients were completely tapered off prednisone; fifteen patients were tapered off prednisone after cycle 2. The dose of administered prednisone decreased by a mean of 59.7% (*p*=3.3x10^–8^) after the first cycle, 87.9% (*p*=2.4x10^–13^) after the second cycle, and 94.6% (*p*=2.2x10^–14^) after the third cycle of rituximab treatment. Among 36 patients who received plasma exchange, there was a statistically significant reduction in plasma exchange sessions after cycles 1, 2, 3 and 4 (Figure 3 (Fig. 3)) (p-values respectively are 0.0029, 0.0008, 0.0021 and 0.0023).

Twenty of the 36 patients required no plasma exchange after 6 months (cycle 1) and in 10 patients, PE was stopped after 12 months (cycle 2) following the initiation of rituximab. In four patients, PE was stopped after rituximab cycle 3. However, two patients out of 36 continued to require plasma exchange. Seven patients showed decreased reduction and further stoppage of mycophenolate mofetil from a maintenance dose of 1.5 g/day. 

**Figure 4:**
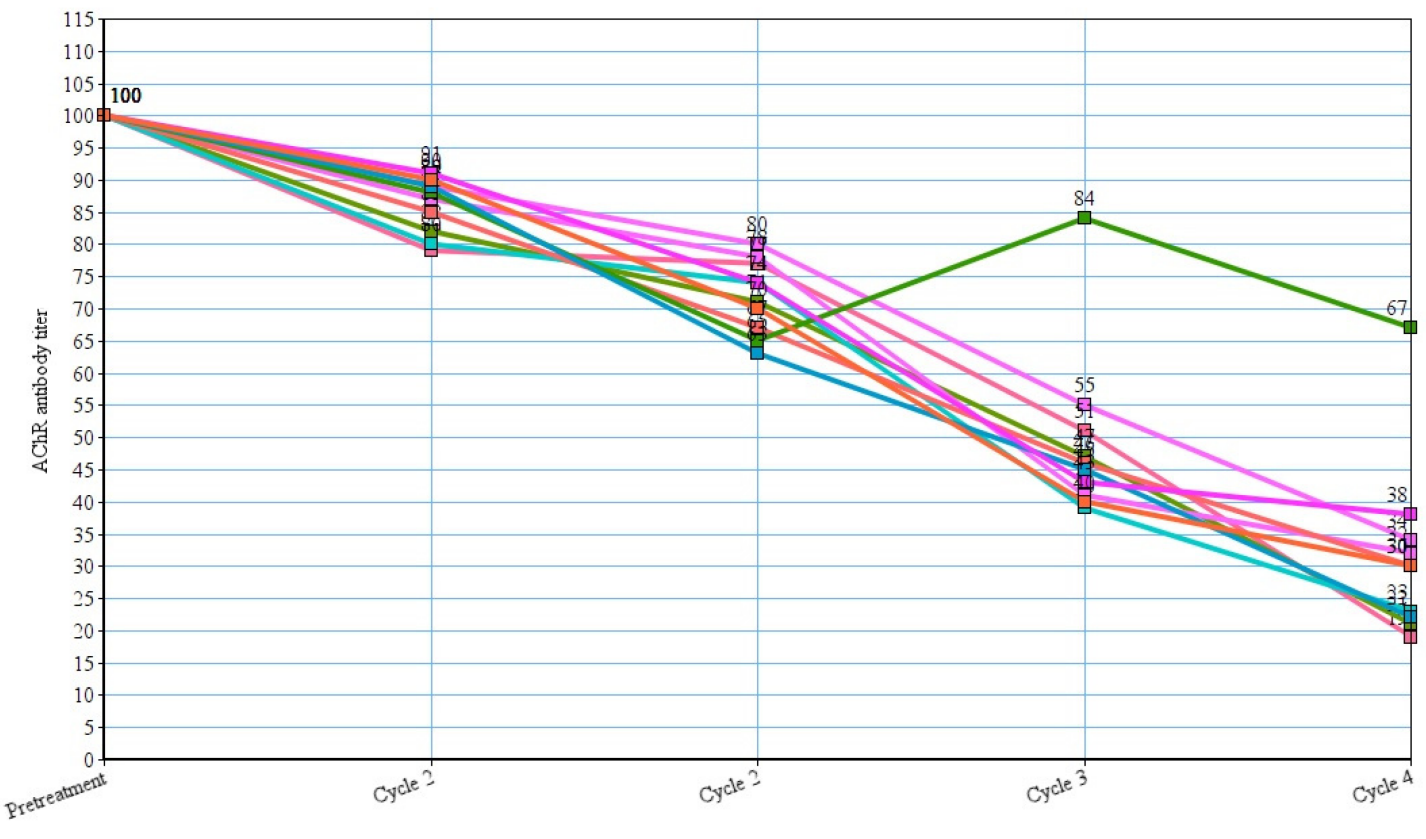
AChR antibody titer reduction after rituximab therapy. A value of 100% was assigned to the titers before treatment with rituximab and expression of data as percent decrement or increment following each cycle of treatment.

In three patients, azathioprine was stopped after 3 cycles of rituximab, but another 5 patients continued to use azathioprine therapy. Out of 10 patients whose AChR antibody titers are available, we observed a marked reduction of titers of AChR antibody with each cycle (p-values respectively were 0.05, 0.049, 0.039 and 0.048) (Figure 4 (Fig. 4)). Fifteen patients reported adverse reactions, but, no deaths occurred due to the adverse effects. 

The most common adverse reactions reported were pruritis and flushing, flushing and shortness of breath and chills/rigors. Three patients developed generalized rash and shortness of breath which was treated symptomatically with chlorpheniramine and hydrocortisone. Four patients developed leucopenia which was later resolved spontaneously. One patient reported chest pain after administration of rituximab, but his ECG, echo and troponin levels were found to be normal. Liver function test was deranged in 5 patients, with aslight raise of SGOT and SGPT, but rituximab was not stopped. All the patients who reported continued to use rituximab either with slow infusion or restarting at a later date.

## Discussion

Our study reported the clinical features of refractory MG patients, based on the inability of reduction of immunotherapy without producing clinical relapse, insufficient response to immunosuppressive therapy, or the development of severe adverse effects to immunosuppressive medications. Statistically significant differences were found in refractory MG patients compared to general MG patients in terms of age of onset of disease, gender proportion, thymoma status, and antibody status, characteristics. 

Our study concurs with previous studies showing that a higher percentage of refractory patients than non-refractory patients have anti-MuSK antibodies. Studies have found that anti-MuSK antibody positive MG patients respond to conventional immunotherapy usually, but require higher corticosteroid doses to manage and remission rates were lower compared to AChR-antibody positive patients [26], [27]. Hence, MuSK-antibody positive MG patients would be more likely to present with refractory disease, according to our findings. 

Many case series of refractory patients as part of larger studies are available online for evaluating the effectiveness of nonconventional therapies for the treatment of refractory disease [28], [29], [30], [31]. Among refractory MG patients, 22 patients (28.94%) have diabetes mellitus. Detailed prevalence reports of association of diabetes mellitus with MG are lacking, but it is estimated to be 2–3% [32], [33]. According to a Taiwanese study, MG cohort had a 1.26-fold increased risk of developing DM compared with the comparison cohort [34]. Our study proves that refractory MG patients are associated with higher risk of diabetes mellitus. 

There are reports describing myasthenia gravis accompanied by DM associated with primary biliary cirrhosis [35], pernicious anemia, autoimmune thyroiditis and autoimmune adrenalitis [36], juvenile chronic arthritis [37], and positive autoantibody for islet cells, gastric mucosa and thyroid gland [38]. Our study showed normal C-peptide and anti-GAD antibodies in all the diabetic patients. But, our study also showed that all the diabetic patients were given steroids. Mean HbA_1C_ was found to be 8.6±2.33. It also indicates poor control of diabetes mellitus in refractory MG patients. 

Our study showed that rituximab led to good to excellent clinical improvement with a reduction of class in MGFA Clinical Classification, complete stoppage of corticosteroid therapy, reduction of co-medication and plasma exchange in refractory MG patients. We also observed substantial statistically significant reduction in dosage requirement of mycophenolate mofetil and azathioprine. We also observed a statistically significant reduction in AChR antibody titers in those patients, whose reports were available. Our study supports the established fact that rituximab is useful in management of refractory MG [39], [40], [41], [42], [43], [44], [45].

Both AChR antibody and MuSK antibody positive patients responded almost similarly to rituximab with reduction of need of immunotherapy and sustained clinical improvement. No difference was noted in thymectomised and non-thymectomised patients regarding effectiveness of rituximab. Rituximab is a humanized monoclonal antibody that binds to CD20 antigen, inducing complement- or antibody-mediated cytolysis [46], [47]. Rituximab attaches itself to one side of B cells containing CD20, forming a cap and drawing proteins over to that side. The presence of the cap changes the effectiveness of natural killer (NK) cells in destroying B cells. When an NK cell finds the B cell with the cap, it has an 80% success rate at killing the cell. But, when the B cell lacked the asymmetric protein cap, it was killed only 40% of the time [48], [49]. 

The effects of rituximab [50] have been described are as follows:

1. The Fc portion of rituximab mediates antibody-dependent cellular cytotoxicity (ADCC) and complement-dependent cytotoxicity (CDC).
2. General regulatory effect on the cell cycle.
3. Increases MHC II and adhesion molecules LFA-1 and LFA-3 (lymphocyte function-associated antigen).
4. Elicits shedding of CD23.
5. Down-regulates the B cell receptor.
6. Induces apoptosis of CD20+ cells.

These combined effects result in the elimination of B cells (normal and abnormal) from the body, allowing new B cells to develop from lymphoid stem cells. 

Rituximab binds to amino acids 170–173 and 182–185 on CD20, which are close to each other as a result of a disulfide bond between amino acids 167 and 183 [51]. Tumor lysis syndrome and renal toxicity are specific to patients using rituximab for hematologic malignancies [52]. Progressive multifocal leukoencephalopathy (PML) after rituximab therapy is also of matter of concern, however, according to a review the relative risk is low [53]. Our study shows that the adverse effects are low. 

The effect of glucocorticoids on metabolism of glucose is likely the result of multiple pathway impairment including beta cell dysfunction (sensitivity to glucose and insulin release) and insulin resistance in other tissues. Evidence for the direct effect of glucocorticoids on beta cell function has been found from cultured rat insulinoma insulin-secreting INS-1E cells [54]. Measurement of impaired insulin release in response to a glucose challenge was demonstrated in prednisone-treated INS-1E cells. The inhibition was reversible in the presence of prednisone with the glucocorticoid receptor antagonist RU486 [54]. The defect may be due to impaired endoplasmic reticulum homeostasis, which in turn may lead to beta cell death.

## Conclusions

We put forward the following observations based on our retrospective analysis. This retrospective study has the limitations in sample size and accurate calculation of incidence. The refractory MG patients are most commonly female with an early age of onset, anti-MuSK antibodies, and thymomas. More research is required for identification of new biomarkers or optimal usage and finding importance of available biomarker tests and response to treatment. Refractory MG patients have higher prevalence and poor control (HbA_1C_ >8%) of diabetes mellitus and dyslipidemia probably due to increased steroid usage. Rituximab is very efficient in treatment of refractory MG with adverse effects being low. 

## Abbreviations

- AChR: Acetylcholine receptor
- ADA: American Diabetes Association
- ADCC: Antibody-dependent cellular cytotoxicity
- AZ: Azathioprine
- BA: bioavailability
- BE: bioequivalence
- CDSCO: Central Drugs Standard Control Organization
- CS: Cyclosporine
- CT: Computerized tomography
- DM: Diabetes mellitus
- GAD: Glutamic acid decarboxylase
- HLA: Human leukocyte antigen
- IQR: Inter quartile range
- IVIG: Intravenous immunoglobulin
- LDLR: Low-density lipoprotein receptor
- LRP4: Lipoprotein receptor-related protein 4
- mAb; Monoclonal antibodies
- MuSK: Muscle specific kinase
- MG: Myasthenia gravis
- MGFA: Myasthenia Gravis Foundation of America
- MTX: Methotrexate
- NK cell: Natural killer cell
- NMJ: Neuromuscular junction
- PE: Plasma Exchange
- PRED: Prednisone
- PYR: Pyridostigmine
- RA: Rheumatoid arthritis
- SLE: Systemic lupus erythematosus
- TA: Tacrolimus
- THY: Thymectomy
- WHO: World Health Organisation

## Notes

### Competing interests

The authors declare that they have no competing interests.

## Figures and Tables

**Table 1:**
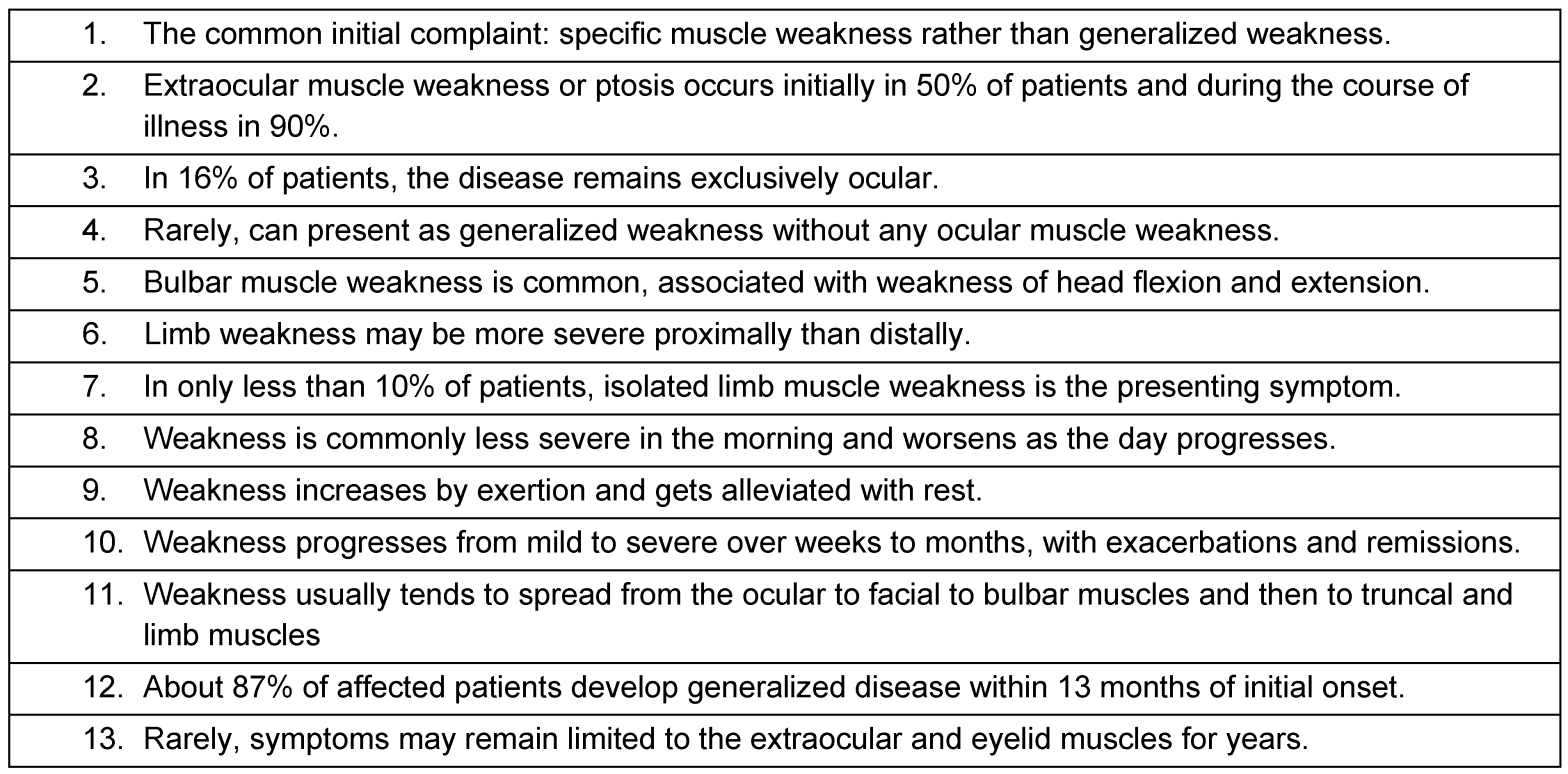
Common clinical characteristics of myasthenia gravis

**Table 2:**
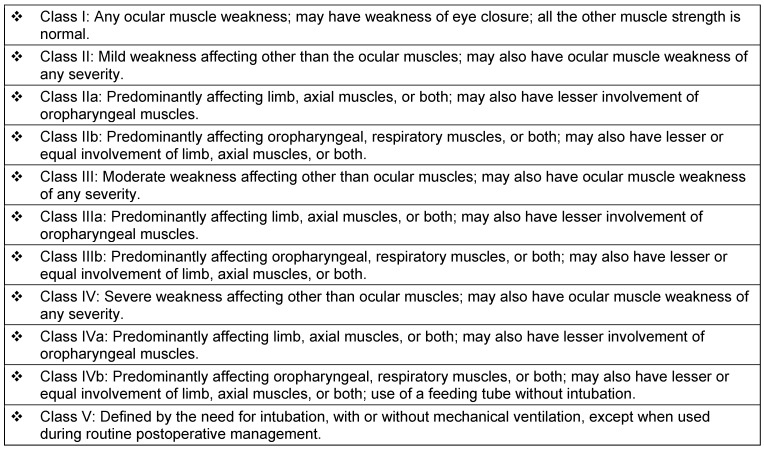
The clinical classification of MG of the Myasthenia Gravis Foundation of America [15]

**Table 3:**
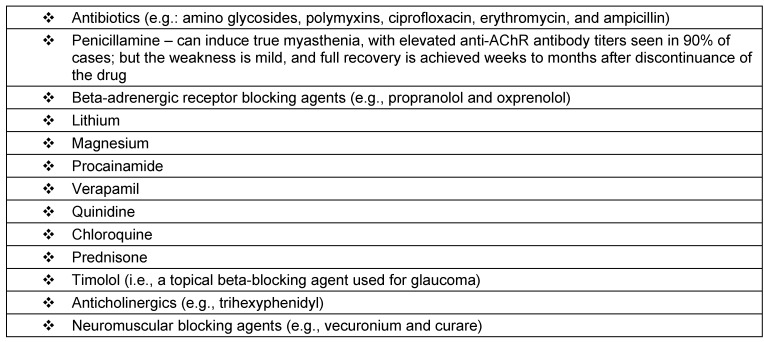
Drugs that exacerbate myasthenia gravis

**Table 4:**
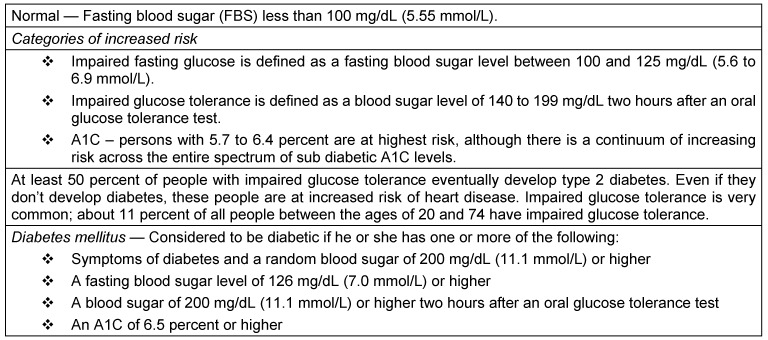
Diagnostic criteria for diagnosis of diabetes mellitus (ADA)

**Table 5:**
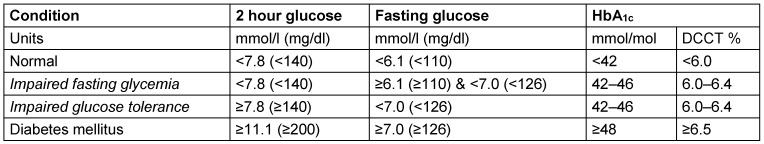
WHO diabetes diagnostic criteria

**Table 6:**
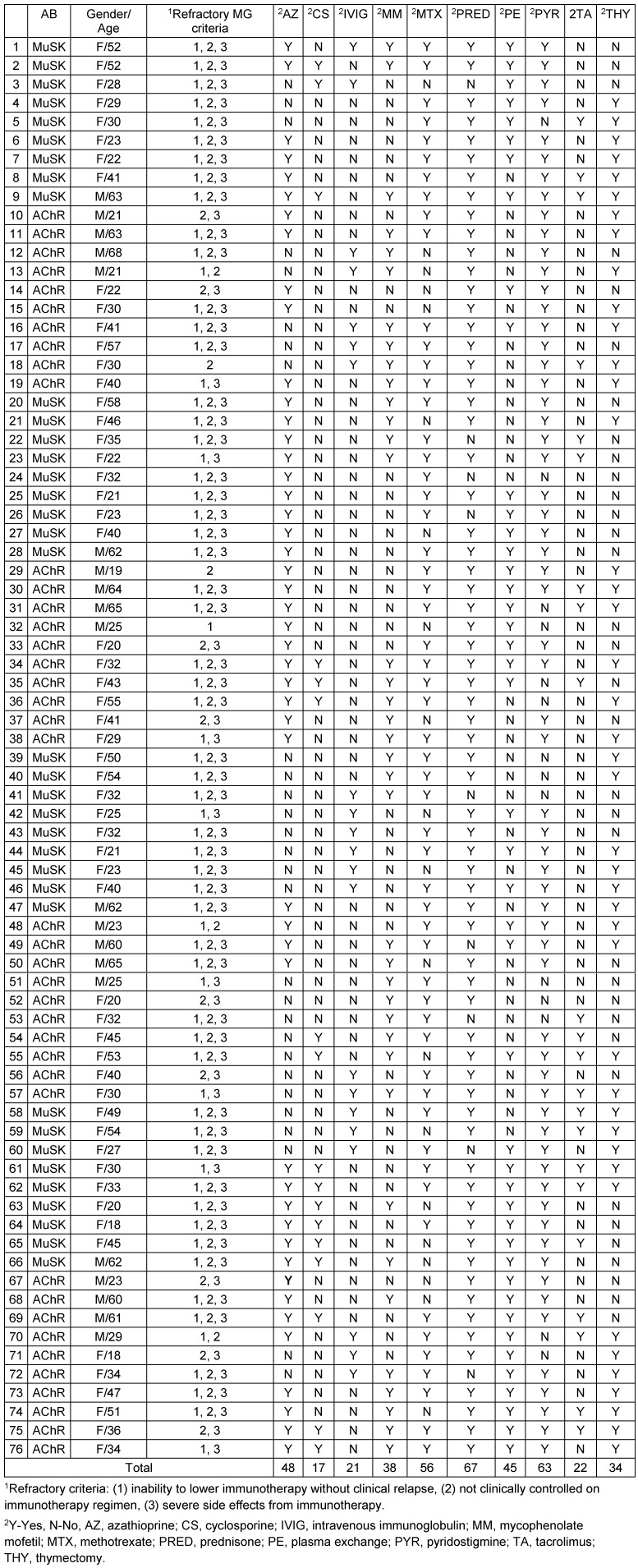
Characteristics of refractory myasthenia gravis patients

**Table 7:**
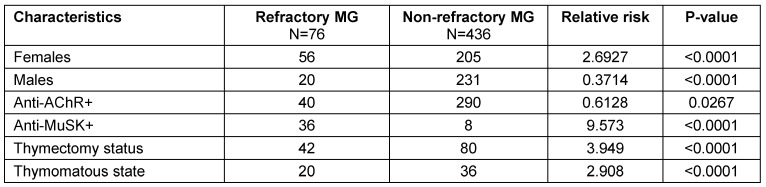
Characteristics of refractory MG and non-refractory MG patients with relative risk

**Table 8:**
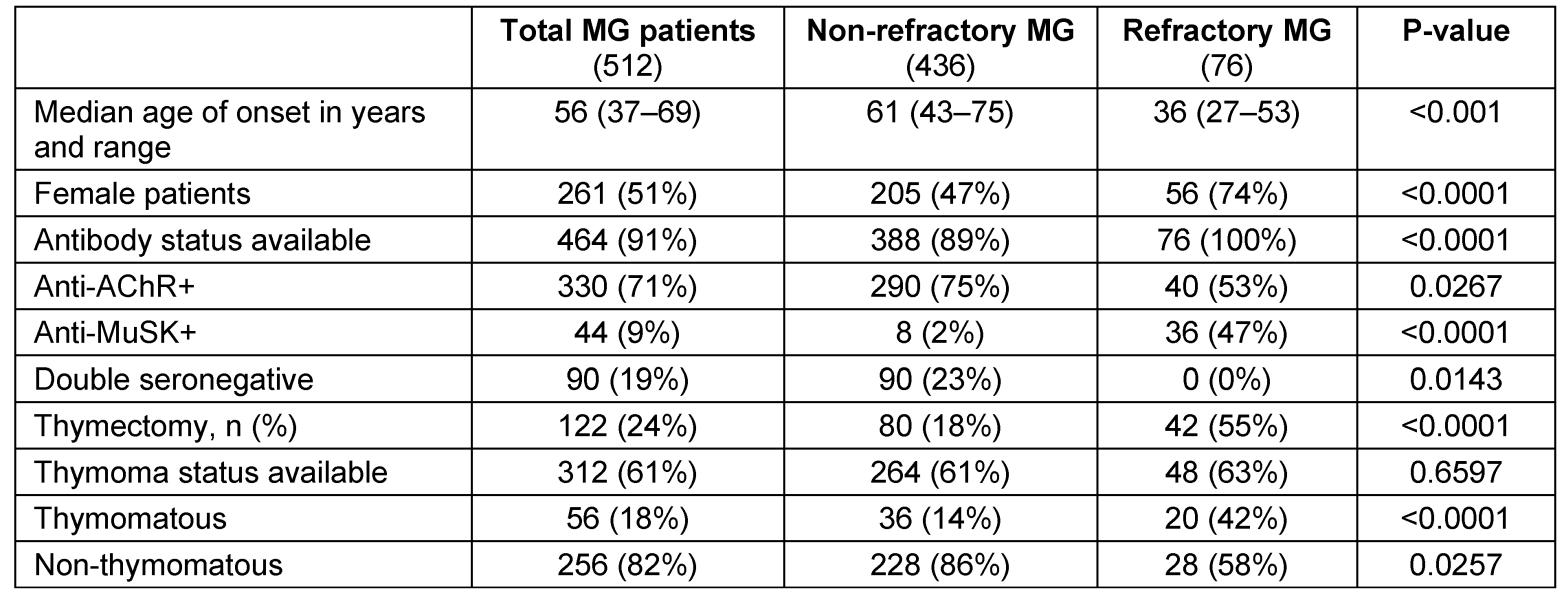
Comparison of MG patients (refractory vs. non-refractory)

**Table 9:**
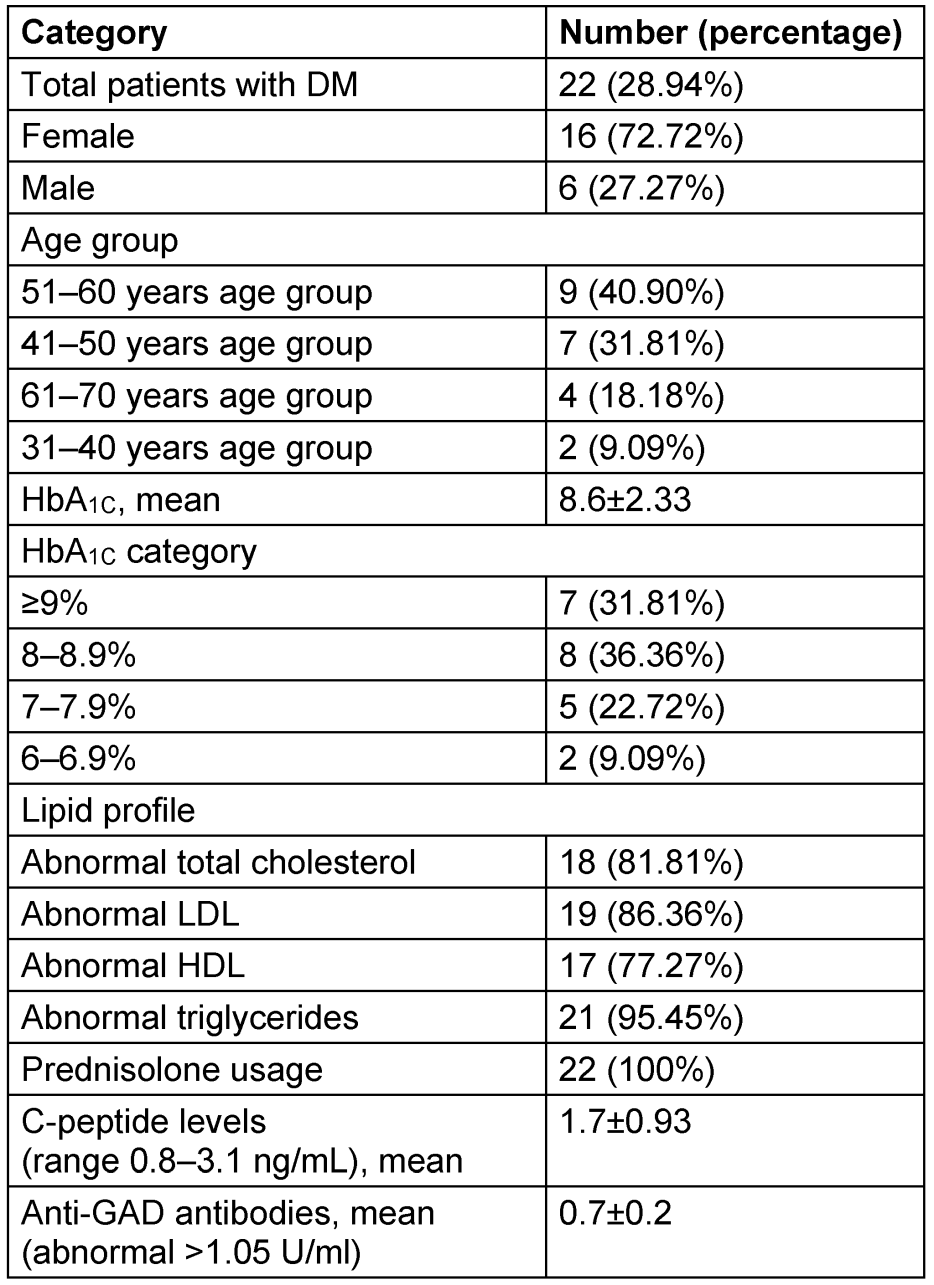
Characteristics of refractory MG patients with diabetes mellitus
